# Management of Osteochondral Lesions of the Talar Dome

**DOI:** 10.2174/1874325001711010743

**Published:** 2017-07-31

**Authors:** Chamnanni Rungprai, Joshua N. Tennant, Ryan D. Gentry, Phinit Phisitkul

**Affiliations:** 1 Department of Orthopaedics and Rehabilitation, University of Iowa Hospital and Clinics, 200 Hawkins Drive, Iowa City, IA, 52242; 2 Department of Orthopaedics, University of North Carolina School of Medicine, 3147 Bioinformatics Building, 130 Mason Farm Road, Chapel Hill, NC 27599-7055, USA; 3 Department of Orthopaedics, Phramongkutklao Hospital and College of Medicine, 315 Ratchawithi Rd., Bangkok, Thailand, 10400

**Keywords:** OLT, OCD, Talus, Arthroscopy, Osteochondral, Cartilage injury

## Abstract

Osteochondral lesion of the talus (OLT) is a common condition associated with ankle injury that brings challenges in the diagnosis and treatment. Symptoms related to this condition are nonspecific including pain, swelling, stiffness, and mechanical symptoms of locking and catching. While the natural history of the OLTs is not well understood, surgical treatment is often required especially in chronic cases and acute cases with displaced articular fragments. Arthroscopic treatment of the OLTs aims to restore ankle joint function and pain relief by the removal of the chondral or osteochondral fragment, debridement and stabilization of cartilage rim and subchondral bone, and stimulate healing of the bone and damaged cartilage. In patients with a large lesion or after a failure of previous bone marrow stimulation, biologic restoration techniques including the use of particulate juvenile cartilage techniques, autogenous chondrocyte implantation, and osteochondral autograft or allograft transplantation may have role. This article summarizes the contemporary concepts in the clinical evaluation and treatment of OLTs with particular emphasis on surgical strategies.

## 1 INTRODUCTION

Osteochondral lesions of the talus (OLT) bring the challenges both of articular cartilage healing and a constrained area of access in the ankle joint. OLTs have been known historically by varied nomenclature, including osteochondritis dissecans, talar dome fracture, transchondral fracture, and flake fracture. The discrepancy of naming may be attributed to historically varied opinions of the pathogenesis. Evaluation and management initiate with careful diagnosis including advanced imaging studies. Conservative treatment methods are usually attempted first. Surgical management has evolved with the advances in ankle arthroscopy techniques, as well as new applications of biologic stimulation and allografting techniques to promote healing of OLTs.

## 2 INCIDENCE

The reported incidence of OLT is based on limited case series in the literature; however, trauma remains the leading causes [1-5]. Bosien *et al.* reported an OLT x-ray incidence of 4% in a series of 121 persistently symptomatic ankle sprains in university students [6]. Additionally several studies have noted the incidence of bilateral OLT to be around 10% [5, 7, 8]. OLT may have higher incidence in the chronic ankle pain patient population, which was noted to be a source of unidentified pathology with delayed diagnosis of OLT in 81% of patients in a single series [9].

## 3 MECHANISM OF INJURY

Ankle sprains and rotational rotational injury have been associated and reported as a potential mechanism of OLT pathogenesis. While the concept of spontaneous osteochondritis dissecans was first applied to the ankle in 1922 [10], a theory of traumatic etiology was popularized by Bernt and Hardy in 1959 [5]. The two theories found some harmony with the 1966 conclusion by Campbell and Ranawat the osteochondritis dissecans creates ischemic pathologic fractures of bone [11].

## 4 LOCATION

Multiple authors have noted that the incidence of medial OLT is higher than that of lateral lesions. Canale reported that all lateral lesions have a history of trauma, while only 64% of medial lesions report trauma [4]. Medial lesions are usually deeper, and more likely to change into cystic lesions, while lateral lesions, shallower, and more likely to have an associated wafer or flake fracture. Historically lesion position was considered to be anterolateral or posteromedial. A recent analysis of 428 ankle MRIs with known OLTs showed 53% of lesions medial and middle, and 26% lateral and middle. The vast majority of lesions were the in the middle (80%), with relatively fewer anterior or posterior (6% and 14%, respectively) [12].

## 5 HISTORY AND PHYSICAL EXAMINATION

Patients with OLT normally present with complaints of pain, swelling, stiffness of the ankle, and in some cases mechanical symptoms of locking and catching. They usually relate the pain to a single injury, recurrent sprains, or feelings of chronic instability [13]. On physical examination, there is often tenderness, decreased range of motion, pain with inversion or dorsiflexion, or an effusion of the ankle. The lack of these signs on physical exam does not exclude an OLT as often the physical exam signs are absent. History and physical exam is often nonspecific and non-diagnostic for OLT requiring further workup with imaging studies [14].

## 6 RADIOGRAPHS

Radiographs are typically the first imaging study performed in evaluation of the ankle. Historically radiographs were considered the principal method of diagnosis of OLT. In 1947 Ray and Coughlin recommended adding oblique projections to the standard anteroposterior and lateral views, noting that the malleoli would hide lesions on the medial and lateral aspect on the standard anteroposterior view [15]. It was later suggested to add a mortise view of the ankle with the ankle in maximal plantar flexion to evaluate lesions along the posterior medial dome [16, 17]. Even with the addition of these views, Verhagen *et al.* found 41% of OLT were missed on radiographic examination [14] which is consistent with other studies [2, 18]. While radiographs are often the initial imaging study, the low sensitivity often warrants further imaging for establishing the diagnosis and the extent of the lesion.

## 7 MRI

MRI was first suggested as an imaging method for known OLT in 1987 but was not recommended for diagnosis at that time [19]. Given its high soft tissue contrast, MRI is able to more thoroughly evaluate the articular cartilage. The characteristic finding of OLT is an area of low intensity on T1 imaging with a signal rim of varying intensity at the junction between the talar bed and osteochondral fragment on T2 imaging [20, 21]. Further studies have shown that MRI is 96% sensitive and 96% specific for the diagnosis of OLT [14] and able to accurately predict stability of the lesion [21, 22]. Given the accuracy of MRI as well as its low risk, it is often considered the most appropriate investigative imaging study after radiographs.

## 8 CT SCAN

Computerized tomography (CT) scanning has been well described in the evaluation of OLTs. In contrast to MRI, CT scans can better define the osseous anatomy. In Verhagen *et al.*’s [14] study, CT was 81% sensitive and 99% specific for the diagnosis of OLT, which showed no statistically significant difference between MRI [14]. It can accurately depict the grade of the lesions, except for early lesions, as these are defined by abnormalities in the articular cartilage which is not seen on CT [23]. The addition of single photon emission computerized tomography (SPECT) imaging to CT adds to the diagnostic value of CT scanning by identifying co-existing pathology as well as showing the activity around the lesion of interest. [24] Recently Tamam *et al.* [25] suggested that SPECT-CT is used in conjunction with MRI for the assessment of OLT. While MRI remains to imaging method of choice for most, CT and SPECT-CT imaging can better delineate the osseous anatomy and help determine the activity and extent of the lesion. For this reason, some now recommend the addition of SPECT-CT for the evaluation of OLT [25].

## 9 OLT STAGING

**Fig. (1):**
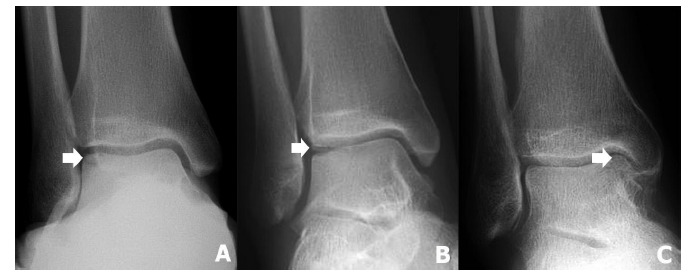
The radiography demonstrates the osteochondral lesions of the talus (OLT). The anterolateral lesions of OLT, Berndt and Harty type 2, 3, and 4, are demonstrated on Figs (**1C**,**1A** and **1B** )(arrow), respectively.

The original radiographic classification system for OLT was developed by Berndt and Harty in 1959 [5] (Table **1** and Fig. **1**) and determined only by plain radiographs. At the time they referred to it as “… an arbitrary classification which was developed to aid understanding of the etiological mechanism of the fracture and to help determine the appropriate treatment [5]. Although radiographs are now rarely used to stage OLTs, the original classification system by Berndt and Harty is still the most commonly used classification system.

**Fig. (2):**
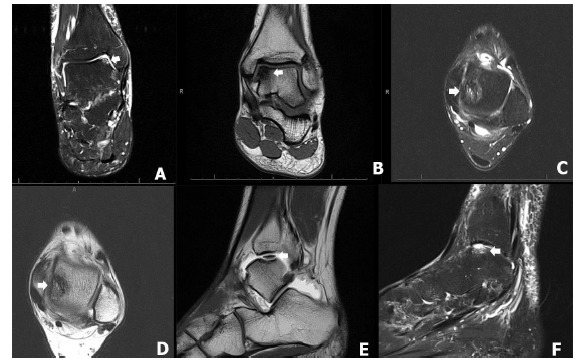
The MRI demonstrates the osteochondral lesions of the talus (OLT). The coronal plane of the MRI demonstrates anteromedial lesions of OLT, Hepple stage 1 and 2A in figure 2A, and 2B (arrow), respectively. The axial MRI demonstrates mid-medial lesions of the OLT, Hepple stage 2A in figure 2C and 2D. The sagittal MRI demonstrates non-displaced mid-medial lesion of OLT, Hepple stage 3, in figure 2E (arrow) and displaced mid-lateral lesion of the OLT, Hepple stage 4, in (Fig. **2F**) (arrow).

Multiple additional classification systems have been proposed based on MRI. The first was described by Anderson *et al.* as a modification of the original Berndt and Harty staging to include a stage 2A, which corresponded with subchondral cyst formation as seen on MRI [5, 17]. Mintz *et al.* showed an 83% correlation in staging classified by MRI integrity of the overlying cartilage and findings at the time of arthroscopy. (Table **2**). When patients were grouped into disease negative (Stage 0-I) or disease positive (Stage II-V), the authors noted a sensitivity of 95% and specificity of 100% [26]. Another commonly used MRI classification system is that reported by Hepple *et al.* [27] (Table **3** and Fig. **2**), which is based on MRI appearance as well as subchondral edema, which was reported as the reason lesions were understaged using previous MRI classifications [26, 28].

The initial staging classification from CT scans was introduced by Ferkel *et al.* [9] (Table **4**). Since that time it has remained the predominant CT staging system. Although useful for defining bony anatomy, it has not been shown to be correlated with patient outcomes [29].

An arthroscopic staging system was first described by Pritsch *et al.* in 1986 and found to have no significant correlation with the preoperative radiographic Berndt and Hardy stage [30]. A second arthroscopic staging system by Ferkel and Cheng [31] was also based on the status of the articular cartilage, but has been shown to be predictive of patient outcomes. Patient with a Grade A-C had better outcomes when compared with Grade D-F as determined by the Modified Weber and AOFAS scoring systems (Table **5**) [29].

## 10 TREATMENT

The natural history of the OLT remains unclear due to paucity of longitudinal follow-up studies. Various treatment strategies have been described for OLT, including conservative treatment (rest/restriction of activities and immobilization in the cast) and operative treatment (excision, excision with curettage, excision with curette and drilling/microfracture, excision with curette and autogenous grafting, excision with curetted and particulated juvenile cartilage, retrograde drilling, autogenous chondrocyte implantation, osteochondral autograft transplantation, and osteochondral allograft transplantation) [32-34]. However, the current literature does not allow firm evidence-based recommendations concerning the treatment to be established [35-37].

## 11 CONSERVATIVE MANAGEMENT

Initially conservative treatment should be considered in the patients who have non-displaced OCD lesion of talus, Berndt and Harty grade I, grade II, and small lesions of grade III [32, 38], or intact cartilage as determined by arthroscopy [15]. However, the current literature does not provide definitive criteria for conservative treatment, which can be initiated in acute, subacute, and chronic injury, up to one year after the onset of injury [32, 39]. The accepted contraindication of non-operative treatment is a displaced intra-articular osteochondral fragment [40].

Conservative management usually consists of rest/restriction of activities [33, 41], non-weight bearing with immobilization in a short leg case [33, 38], and with or without the use of NSAIDs [38] for 3 weeks to 4 months [4, 42-44], followed by progressive weight bearing in the CAM boot with physical therapy for 6-10 weeks [32, 38]. The purpose of conservative treatment is to decrease or unload the injured cartilage, so bone edema can be resolved and necrosis can be prevented. Another objective is to allow the detached fragment to heal to the underlying and surrounding bone [45]. However, based on current literature, there is no specific conclusion regarding duration of non-operative treatment, method of immobilization, weight bearing status, the use of NSAIDs, and physical therapy protocol [13].

The functional outcomes after conservative treatment of OLT have been described in the literature with variable methods and results [4, 16, 30, 41-44, 46, 47]. There were good/excellent outcomes approximately 59 percent after rest/restriction of activities without immobilization [16, 30, 41, 44, 48, 49], while the good/excellent outcomes were 41 percent with immobilization [2, 4, 41-44, 47-49]. While the natural history of OLTs is not well described, Klammer *et al.* reported no increase in symptoms in 86% (43/48 ankles) of minimally symptomatic OLTs treated nonoperatively, followed at least two years with MRI [6]. Recently, meta-analysis by Tol *et al.* demonstrated that the overall good/excellent results were 45 percent (91 of 201 patients) of the patients who had OLT and treated by conservative management; however, the truly successful rate of conservative treatment of OLT is still debatable [39]. In addition, few studies reported that arthritis of the ankle joint has been observed in approximately 50 percent of the patients who were treated with conservative treatment [4] but it could not be determined whether operative intervention might prevent the degeneration of the ankle joint [4]. McCullough *et al.* studied a small case series (n = 10) with an average follow-up of 15 years and 11 months and found that OCD lesions may not heal over many years, but ankle joint were relatively asymptomatic and arthrosis was minimal [41]. In addition, no radiographic difference of arthritis was noted between the patients who underwent conservative versus operative procedure for OLT treatment.

## 12 PROGNOSTIC FACTORS

Several current studies exist that demonstrate the prognostic factors for the operative treatment of the OLT. There have been multiple studies addressing different patient factors and lesion characteristics that may yield a poorer outcome. Lesions smaller than 15 mm [50-53], contained lesions [54], and anterolateral lesions [54] are considered to be the positive prognostic indicators; negative indicators include older age (> 33-40 years old) [51, 55], lesions deeper than 7 mm [55-57], lesions larger than 15 mm [51], cystic lesions [56], medial talar lesions [55], higher BMI, history of trauma, longer duration of symptom, and presence of osteophytes [50].

## 13 OPERATIVE TREATMENT

The principle of operative treatment is to restore ankle joint function and pain relief by the removal of the chondral or osteochondral fragment, debridement and stabilization of cartilage rim and subchondral bone, and stimulate healing of the bone and damaged cartilage. Curettage, drilling, or microfracture techniques are recommended for OLTs which are no larger than 15 mm and no deeper than 7 mm [50, 51, 57]. On the other hand, the large lesions or lesions failed from primary bone marrow stimulation surgery should be considered for autologous chondrocyte implantation (ACI), osteochondral autograft transplantation (OATs or mosaicplasty), osteochondral allograft transplantation, or metal inlay implant [13, 34, 58].

Indications for operative treatment include failure of conservative treatment for at least period of 6 weeks to 6 months [50] and acute osteochondral fragment Berndt and Harty grade III and IV [4, 42]. However, there is no current literature providing definitive timing for operative treatment. O’ Farrell *et al.* recommended that the best timing of surgery is within 12 months after an inciting injury [59]; however, Alexander *et al.* demonstrated that delaying surgery several months (range, 3 to 36 months) did not affect the outcomes of operative treatment [60]. Postoperative protocols vary based on the specific treatment methods but generally involve early range of motion followed by protected weightbearing in a boot and gradual return to sports.

## 14 POSITIONING

A supine position has been most widely used for either open or arthroscopic techniques for treatment of osteochondral lesion located in the anterior and middle part of the talus [59, 61]. Lateral decubitus position has been used for open approach for lateral lesions [62]. Prone position has been reported for the arthroscopic treatment for the osteochondral lesion located on the posterior part of the talus [63-65]. 

## 15 SURGICAL APPROACH

### 15.1 Open Technique

The anterolateral and mid-lateral lesions of the talus can be accessed either open or anterior arthroscopic techniques [61, 66]. The open technique can be performed using anterolateral approach [2], anterior arthrotomy of the ankle joint [59], or fibular osteotomy [67, 68] while the posterolateral lesion can be accessed from posterior ankle arthroscopy [69] or open posterolateral approach [67, 70].

The anteromedial and mid-medial lesions of talus can be accessed either anterior ankle arthroscopy [61, 66] or open techniques [67]. The open technique can be performed using anterior ankle arthrotomy [59] or open technique through medial malleolar osteotomy [2, 59, 67] while the lesions on the posteromedial and posterior parts of the talus can performed using posterior ankle arthroscopy [69], medial malleolar osteotomy [67, 71], or open posteromedial approach.(67, 71)

### 15.2 Arthroscopic Technique

Standard anteromedial (AM) and anterolateral portal (AL) have been used for OLTs with ankle distraction.Figs. (**3A** and **3B**) [62, 66, 72]. The anteromedial accessory portal can be established if needed to access lesions on the medial talar dome. Fig (**3B**). In addition, posterolateral (PL) and posteromedial (PM) portals can be used for lesions located in the posterior half of the talus, as 54 percent of the talar dome can be visualized and accessed through the posterior ankle arthroscopy [63-65, 73].

## 16 SURGICAL STRATEGIES

### 16.1 Excision

The loose osteochondral fragment removal can alleviate ankle pain by preventing loose body irritation or blocking and preventing further damage by the loose osteochondral fragment to the normal cartilage tissue. Previous studies with limited number of patients have demonstrated that the overall success rate of excision of OLT varied from 33 to 92 percent after using open techniques [47, 59, 74].

### 16.2 Bone Marrow Stimulation

### 16.3 Excision and curettage

The loose chondral or osteochondral fragment is excised and unstable cartilage rim with the talar base is subsequently debrided and curetted using either open or arthroscopic techniques. The previous literature reported that the overall success rate of excision and curette was 63 percent (119 of 189 patients); however, the success rate varies between 47 to 89 percent from 9 studies [4, 30, 42, 43, 62, 66, 72, 75, 76]. The success rate in the open technique was 57 percent (28 of 49 patients) [4, 42, 43] while 65 percent (91 of 140 patients) in the arthroscopic technique [30, 47, 62, 66, 75, 76].

### 16.4 Excision, Curette, and Microfracture/Drilling

**Fig. (3):**
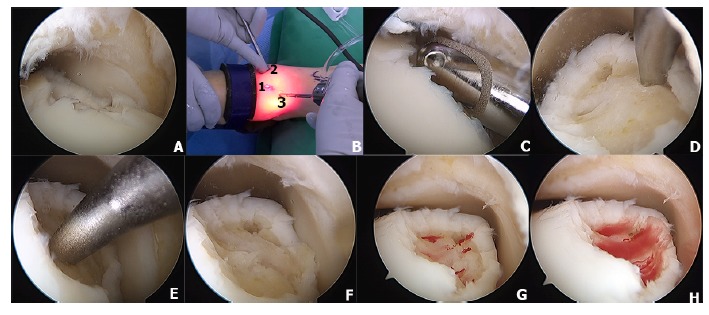
A microfracture technique of osteochondral lesion of the talus is demonstrated. A mid-medial osteochondral lesion of the talus is demonstrated on Fig. (**3A**). Anterolateral (2), anteromedial (1), and accessory portals (3) are demonstrated on Fig. (**3B**). The unstable cartilage rim was debrided using 4-mm shaver (**3C**). The chondral pick is used to create microfracture holes approximately 4-mm apart (3D-3E) and the base of the osteochondral lesion after complete debridement and microfracture (**3F**). After the tourniquet was deflated, the bleeding from the microfracture holes are demonstrated in Fig (**3G**-**3H**)

The microfracture technique induces the repair of cartilage damage with fibrocartilage, resulting in good functional outcomes as reported in the previous studies [34, 50, 61, 78-80]. Most of lesions in the anterior and middle lesions of the talus could be treated arthroscopically through standard portals and with awls or chondral picks of different angles Figs. (**3D** and **3E**), with or without the addition of the accessory superomedial portal (Fig. **3B**) [61]. Multiple small holes are created in the subchondral plate at 3- to 4-mm intervals (Figs. **3D** and **3E**) stimulating the release of mesenchymal stem cells, growth factors, and healing proteins [81]. (Figs. **3F**-**3H**). The coagulation response results in the formation of a fibrin clot (Fig. **3H**) leading to the eventual formation of fibrocartilaginous repair tissue mainly type I collagen [34]. In addition, microfracture technique has proven to be effective for treatment of both chondral and osteochondral lesions of the talus [79]. Indication for microfracture is recommended as a first-line treatment, especially in osteochondral defects of the talus measuring less than 1.5 cm^2^ [57, 80]. However, the major disadvantage of the microfracture technique is that the durability of fibrocartilage tissue is inferior to normal hyaline cartilage [58].

The success of treatment of OLT with microfracture techniques has been reported in the literature [50, 61, 78, 79, 82, 83]. Beacher *et al.* studied in 45 patients with arthroscopic debridement and microfracture with improvement of functional outcomes (31 of 39 patients had excellent/good Hannover scoring system) and pain relief (mean pre-operative VAS 6.5/10 improved to 2.4/10 post-operatively) at an average follow-up of 5.8 years [78]. Chuckpaiwong *et al.* studied 105 patients with anterior ankle arthroscopic debridement and microfracture for OLT. They reported that microfracture technique demonstrated significant improvement of functional outcomes as measured with VAS and AOFAS scores (mean pre-operative VAS 8.2/10 improved to 3.8/10 post-operatively and mean pre-operative AOFAS scores 42 improved to 68 points at 12 months post-operatively) [50]. They also reported the improvement of functional outcomes in patients who had failed primary microfracture and underwent repeated microfracture in 17 patients (mean improvement of AOFAS scores was 17.2 points) [50]. Moreover, MRI was used to evaluate OLT after arthroscopic microfracture in 22 patients by Kuni *et al.* They demonstrated significant reduction of the defect size and significant improvement of the functional outcomes (mean AOFAS score was 87.5 points post-operatively) [83].

More recently, comparative study has demonstrated the outcomes of the marrow stimulation surgical techniques. Gobbi *et al.* performed a prospectively randomized controlled trial (Level II study) with 33 ankles in 32 patients, showing no difference between chondroplasty, microfracture, and OAT technique with regard to Ankle-Hindfoot Scale (AHS) and the Subjective Assessment Numeric Evaluation (SANE) rating in patients with OLT [80].

The purpose of drilling technique is similar to the microfracture technique. After excision and curettage of the osteochondral lesion, drilling stimulates the bone marrow locally to produce fibrocartilagenous repair tissue to cover the osteochondral defect [35]. The successful treatment of OCD lesion with drilling has been reported in the previous studies [60, 77]. Alexander *et al.* reported 89 percent of the patients (16 of 18 patients) having good/excellent long-term outcomes with an average follow-up of 58 months [60]. However, Robinson *et al.* studied 22 patients with excision, curette, and antegrade drilling and reported only 31.8 percent (7 of 22 patients) of the patients having good outcomes. Two patients reported persistent medial ankle pain after transmalleolar drilling [77].

### 16.5 Excision, Curette, and Particulated Juvenile Cartilage

**Fig. (4):**
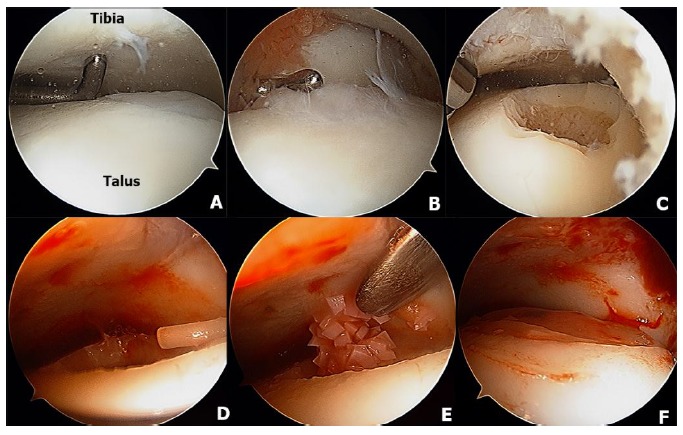
A particulated juvenile cartilage technique has been demonstrated in the Fig. (**2**). A mid-medial talar lesion is demonstrated in the Figs. (**4A**-**4B** and **4C**) is demonstratesed OLT after complete debridement (2C) then the fibrin glue was applied on the base of the lesion (4D) and the particulated juvenile cartilage was introduced into the defect (4E). The fibrin glue was subsequently laid on top of the juvenile cartilage (4F).

Particulated juvenile articular cartilage is prepacked allograft from donors aged younger than 13 years with viable human cartilage cells. The DeNovo NT (natural tissue) graft (Zimmer, Warsaw, IN) is a relatively new product that uses scaffold-free allogenic juvenile cartilage that can be implanted into a defect and secured with a fibrin sealant [84]. This technique has the potential to replace hyaline cartilage architecture without donor site morbidity [85]. Particulated juvenile cartilage can be performed through either open or arthroscopic techniques without malleolar osteotomy because it does not require perpendicular access to the lesion as is often needed for autograft or allograft transfer [86]. However, if the lesions are unreachable, malleolar osteotomy can be performed if needed. The surgical technique of particulated juvenile cartilage has been demonstrated in (Fig. **4**). Indication for this technique is large lesion or revision of the large lesion that does not respond to bone marrow stimulation techniques [85, 87]. 

Previous studies have reported significant improvement of functional outcomes and pain relief after performing this technique in patients who had failed primary bone marrow stimulation treatment [84, 85, 88]. Coetzee *et al.* studied in 24 ankles with particulated juvenile cartilage, reporting 78 percent of the patients having good or excellent outcomes. Improvement of functional outcome (mean AOFAS scores were 85 and mean FAAM activity and sport were 82 and 63 post-operatively) and pain relief (100-mm VAS = 24) was demonstrated with an average follow-up of 16.2 months. One partial graft delamination had been reported at 16 months but no major complications were reported in their study [85].

### 16.6 Retrograde Drilling (RD)

Retrograde drilling (RD) has been used in patients who have intact cartilage as it can avoid injury to the undetached talar cartilage as a non-transarticular procedure. Previous studies have reported the improvement of functional outcomes in both juvenile and adult population [89-97]. Taranow *et al.* first described this technique in 1999 through anterolateral and posterolateral approaches [90]. They studied 16 patients and reported that the mean AOFAS score was 53.9 pre-operatively and improved to 82.6 at 25 months post-operatively. In addition, Kono *et al.* also demonstrated that RD technique yielded significant improvement in AOFAS scores from 70.2 pre-operatively to 96.8 at 2-year post-operatively [91]. Moreover, Ander *et al.* reported significant pain relief from 7.5/10 pre-operatively to 3.7/10 at 29-months post-operatively and the subjective function increased from 4.6/10 pre-operatively to 8.2/10 at 29-months post-operatively [92].

Due to the technical demands of accurate drilling [93] with a previous study reporting 20 percent failure rate [94], several techniques has been developed in order to improve the accuracy and decrease operative time. MRI guidance has proved to be helpful in a variety of percutaneous musculoskeletal procedures, and Kerimaa *et al.* demonstrated that MRI guidance has 100 percent accuracy, is safe, and technically feasible with no complications for retrograde drilling of OLTs [95]. In addition, Hoffmann *et al.* reported that an electromagnetic navigation system (ENS) demonstrated higher accuracy and shorter operative time compared to standard fluoroscopic technique [93]. Recently, navigated retrograde drilling has been introduced by Richter *et al.* who used navigated retrograde drilling to treat 52 patients with OLT. 48 patients were available to analyze at the post-operative visit with improvement of functional outcomes (mean SF-36 of 96 points (range, 86-100 points) and mean VAS score of 93 points (range, 86-100 points) at the final post-operative visit) [96].

### 16.7 Excision, Curette, and Autogenous Bone Grafting

The purpose of autologous bone graft is filling the OLT defect and restoring the weightbearing properties of the transchondral lesions of the talus after excision and curettage [98]. Bruns *et al.* [99, 100] and Draper *et al.* [98] reported that the overall success rate of this technique was 85 percent (28 of 33 patients). Bruns *et al.* studied 26 patients in 1993 and reported good results in 70 percent of the patients.(100) In addition, Draper *et al.* performed a retrospectively comparative study between excision, curette, and drilling (17 patients) versus autologous grafting (14 patients) and they demonstrated that autogenous bone grafting group had significantly better overall outcome scores, ankle range of motion, less pain, and with restoration of subchondral bone on radiography compared to drilling group. They concluded that bone grafting of the lesions yields better long-term clinical outcomes compared to curettage plus drilling [98].

### 16.8 Fixation of the Osteochondral Fragment

The previous studies have reported the successful treatment of large osteochondral lesions by internal fixation [32, 47, 101, 104]. The lesion usually occurs in the anterolateral part of the talus and is associated with trauma [47]. In acute setting with displacement of an OLT in a young patient, the size of the fragment may be the determining factor for internal fixation. DeLee *et al.* suggested internal fixation of the lesion if it is larger than one third of the size of talar dome [103] while Stone *et al.* recommended fixation of the lesion larger than 7.5 mm [71]. The largest series has been reported by Kumai *et al.* who studied in 27 patients with mean follow-up of 7 years, with 89 percent of the patients (24 of 27 patients) reporting good outcomes [101]. In addition, Chandran *et al.* reported excellent results after fixation of inverted OLT using three absorbable pins in 2008 [102]. Schepers *et al.* also reported successful treatment of inverted OLT fragment with the internal fixation with bioabsorbable pins in 2011 [102]. Moreover, Kim *et al.* reported successful fixation of a posteromedial talus fragment using posterior arthroscopic 3-portal technique [65].

### 16.9 Autologous Chondrocyte Implantation (ACI)

Autologous chondrocyte implantation (ACI) involves the use of cultured chondrocytes with a periosteal patch. The cartilage cell can be harvested from the detached cartilage, the margins of the lesion, or from the knee joint [105]. The surrounding cartilage and the subchondral bone defect require debridement until the cartilage rim is stable and no necrotic tissue remains on the osteochondral base. The harvest typically yields a 200- to 300-mg specimen of cartilage and the cells are cultured for 6 to 8 weeks, resulting in 12 million cells available for implantation, at least a tenfold increase [106]. The ability to culture large numbers of cells for implantation is critical to the success of the procedure [107]. A second-stage procedure is performed at 6 to 8 weeks after the cells have been cultured. An anterolateral lesion may be approached without an osteotomy, while a posteromedial lesion requires a medial malleolus osteotomy. The lesion is measured and templated, and periosteal tissue is obtained from the tibia. Fibrin sealant and sutures are used to secure the periosteal patch to the articular surface, and the cultured cells are injected under the patch [106]. Indication for the surgery includes focal full-thickness contained defect or small contained osteochondral defect of the talus (greater than 1.0 cm^2^ - 1.5 cm^2^) [108, 109].

Several previous studies reported the successful treatment of a focal chondral defect using ACI technique with the mean improvement of AOFAS score from 25.2 to 45.0 [105, 108-112] Whittaker *et al.* studied 9 patients with an average of 23 months follow-up with mean improvement of the Mazur ankle score of 23 points with minor morbidity at the donor site [108]. All defects were filled, and stable cartilage and biopsy showed mostly fibrocartilage with some hyaline cartilage [108]. In addition, Baums *et al.* studied 12 patients with mean follow-up of 63 months, reporting that the improvement of AOFAS score was 43.5 to 88.5 and Hanover score was improved from 40.4 to 85.5 at final post-operative visit [110]. Giannini *et al.* performed ACI on 46 patients, with an average follow-up of 18 months [105, 113]. The AOFAS score was improved from 57.2 pre-operatively to 89.5 at 36 months post-operatively [105]. Recently, matrix associated ACI (MACI) has emerged replacing the need for periosteal flap and thereby providing easier and time-saving handling [114]. Ander *et al.* reported their result with MACI in 22 patients with mean follow-up of 63.5 months. The technique demonstrated significant increase AOFAS score from 70.1 to 95.3, VAS score from 5.7/10 to 0.9/10, and Tegner activity level improved from 2.4 to 4.7 at 60 months follow-up with no major complications [109]. Promising results have been reported from the ACI techniques; however, the donor site morbidity and highly cost remain potential barriers.

### 16.10 Osteochondral Autograft Transplantation

This technique is a reconstructive bone grafting technique that use one or more cylindrical osteochondral grafts from the less weightbearing periphery of the ipsilateral knee [115, 116] or ipsilateral talus [117] with transplantation into the prepared defect site on the talus [118]. The aim of osteochondral autograft transplantation is to reproduce the mechanical, structural, and biochemical properties of the original hyaline articular cartilage which was injured and has impaired function [117]. Indications include a large osteochondral lesion (greater than 10-15 mm) or a large lesion after failure of other previous mentioned techniques [116, 117]. The techniques that can be performed include either mosaicplasty [67, 116, 119] or osteochondral autograft transplantation (OATS) procedures [115, 117, 118].

Osteochondral autograft transplantation has been proven to be a successful method with significant improvement of functional outcomes and the mean AOFAS scores were improved 5.7 to 27 points post-operatively [67, 115-122]. Hangody *et al.* studied 11 patients who underwent mosaicplasty autogenous osteochondral technique through arthrotomy of the ankle joint in 1997, with 82 percent of the patients reporting good results using Hannover Ankle/Bandi Knee Morbidity Scores [116]. In addition, Scranton *et al.* reported 90 percent of patients (45 of 50 patients) having good/excellent results and satisfied with the surgery with mean average of follow-up of 36 months [123]. Moreover, Sammarco *et al.* studied 12 patients after using ipsilateral talar autograft, reporting significant improvement of AOFAS scores from 64.4 to 90.8 at a follow-up of 25.3 months [117].

Although the promising results have been reported in the previous studies, this technique can violate the normal cartilage in asymptomatic joint and has associated significant donor site morbidity [119]. Knee pain was reported in 5-50 percent after using autograft from the knee joint [117-119, 121, 125-126]. In addition, Reddy *et al.* reported 36 percent of the patients (4 of 11 patients) having poor knee outcomes as measured with Lysholm criteria [127]. The potential complication of chronic knee pain should be explained during preoperative counseling to the patients.

### 16.11 Osteochondral Allograft Transplantation

**Fig. (5):**
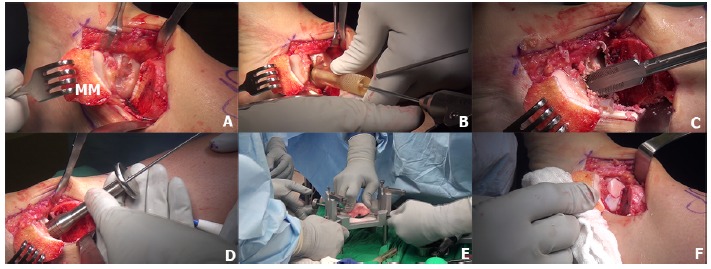
The fresh osteochondral allograft transplantation technique is demonstrated. Medial malleolus osteotomy is performed to access the large lesion on the mid-medial of the talus (5A, MM = medial malleolus). The lesion was measured by cannulated allograft OATS sizers and the K-wire was inserted on the center of the lesion (5B) and the counterbore is then drilled to the lesion and subchondral bone to a depth of 6- to 8-mm (5C). The sizer is used to measure the depth (5D) and the donor fresh talar allograft was secured in the allograft OATS workstation (5E). The talar allograft is implanted into the recipient talar bed until all edges are flush with the surrounding cartilage rim (5F).

Osteochondral allograft transplantation is an effective procedure for the treatment of large OLTs using mature hyaline cartilage allograft which provides an anatomically articulating surface and prevents ankle joint arthrosis by avoiding excessive weight on the remaining portion of the talus [128, 129]. Allograft tissue transplantation is appropriate for large osteochondral defects where duplication of the anatomy would be difficult or impossible with autogenous bone graft [129]. In addition, this technique eliminates the donor site morbidity and the single graft use can minimize the fibrocartilagenous ingrowth as a consequence of the multiple graft use in the autograft mosaicplasty [130]. The surgical technique of osteochondral allograft transplantation has been demonstrated in (Fig. **5**). The indication for the osteochondral allograft transplantation includes large and deep osteochondral defects and failure of previous surgical treatment [129, 131, 132]. 

Previous studies have reported that osteochondral allograft transplantation demonstrates significant improvement of functional outcomes [129, 132-137]. Berlet *et al.* performed fresh osteochondral allograft in 19 patients with an average follow-up of 3.3 years. They reported improvement of AOFAS score from 61 pre-operatively to 79 post-operatively; however, there was no improvement in SF-36 PCS and MCS subscale [132]. In addition, El-Rashidy *et al.* also studied 42 patients with osteochondral allograft transplantation with an average follow-up of 37.7 months. The AOFAS and VAS scores were improved from 52 to 79 and 8.2 to 3.3 post-operatively. They also demonstrated that the allograft was stable, congruous, and had minimal subsidence after repeated MRI at final post-operative visit [133]. Moreover, Adams *et al.* reported the mid-term results with an average follow-up of 48 months after performing fresh osteochondral allograft. They reported significant pain relief (mean VAS was 6/10 pre-operatively and improved to 1/10 post-operatively) and mean post-operative AOFAS scores were 84 at final follow-up. Post-operative radiography demonstrated lucencies at the graft-host interface in five patients, but there was no graft failure [134]. Furthermore, Raikin *et al.* studied 15 patients with allograft transplantation, and they demonstrated significant improvement of the mean AOFAS scores from 38 to 83 and VAS from 8.5/10 to 3.3/10 at 54 months post-operatively. There were no complications reported in their study; however, the radiographic analysis revealed some evidence of collapse or resorption of the graft in ten of the fifteen ankles and nine ankles demonstrated decrease joint space overlying the graft area [135].

### 16.12 Metal Inlay Implant

A novel metal inlay implant (Hemicap, Arthrosurface, Frankin, MA) technique was developed in 2007 for the lesion on medial side of the talus. The purpose of this implant is to reduce pain and prevent further cystic formation at the OLT [138]. This technique requires precise surgical technique because a protruding implant can injure the adjacent tibial plafond, while deepening of the implant leads to collapse of surrounding cartilage and subchondral bone [138]. Indication for this implant includes the patients who have localized medial talar dome with large defect after failed primary surgery [139].

There is a currently limited literature to demonstrate the short-term and long-term outcomes of this technique. The only available study is the prospective cohort by van Bergen *et al.* who studies 20 patients. They demonstrated that the mean of AOFAS score improved from 62 to 87, and SF-36 (PCS subscale) improved from 36 to 45 at an average follow-up of 3 years, with no significant improvement of the SF-36 (MCS subscale) [139, 140].

## 17 SUMMARY

- The etiology and mechanism of injury of the OLT remain unclear; however, it seems to be associated with either acute ankle injury/fracture or chronic ankle instability.
- Although the standard treatment of the OLT has remained controversial, a non-displaced Berndt and Harty Grade I and II lesion should be initially treated with conservative treatment with immobilization and restriction of the activity. If conservative treatment fails, surgical treatment is indicated.
- An acute, displaced osteochondral lesion of the talus which is larger than 7.5 mm or larger than one third of the talar dome in young patients can be successfully treated with an open reduction and internal fixation of the fragment.
- If the size of the lesion is not larger than 15 mm or deeper than 7 mm, bone marrow stimulation technique including excision, curettage, and drilling or microfracture can be performed *via* anterior or posterior arthroscopic technique. Even though the bone marrow stimulation techniques have been proven to be effective treatment for symptomatic patients with small osteochondral lesions of the talus, the reparative tissue forming after the bone marrow stimulation is fribrocartilage (Type 1 collagen predominant) with less durability compared to normal hyaline cartilage (Type 2 collagen predominant).
- If the size of the lesion is larger and deeper or the sequela of failed previous bone marrow stimulation techniques, there is a good evidence to support the use of particulate juvenile cartilage techniques, autogenous chondrocyte implantation, and osteochondral autograft or allograft transplantation. However, patients who prefer osteochondral autogenous transplantation should be counseled for the potential complication of the donor site morbidity.

## Figures and Tables

**Table 1:** Radiographic classification of transchondral fractures of the talus.

**Stage**	**Definition**
**I**	Compression fracture with intact overlying cartilage
**II**	Incomplete avulsion of an osteochondral fragment
**III**	Complete avulsion of an osteochondral fragment without displacement
**IV**	Avulsed fragment displaced into joint

Berndt AL, Harty M. Transchondral fractures (osteochondritis dissecans) of the talus. J Bone Joint Surg Am. 1959;41-A:988-1020.

**Table 2:** MRI grading system with arthroscopic correlation for osteochondral lesions of the talus.

**Stage**	**Definition**
**0**	Normal Cartilage
**I**	Abnormal cartilage signal but intact
**II**	Fibrillation or fissures in cartilage not extending to bone
**III**	Cartilage flap present or bone exposed
**IV**	Loose nondisplaced osteochondral fragment
**V**	Displaced osteochondral fragment

Mintz DN, Tashjian GS, Connell DA, Deland JT, O' Malley M, Potter HG. Osteochondral lesions of the talus: a new magnetic resonance grading system with arthroscopic correlation. Arthroscopy. 2003;19(4):353-9.

**Table 3:** MRI staging system for osteochondral lesion of the talus.

**Stage**	**Definition**
**1**	Articular cartilage damage only
**2a**	Cartilage injury with underlying fracture and surrounding bony edema
**2b**	Stage 2a without surrounding bony edema
**3**	Detached but nondisplaced fragment
**4**	Detached and displaced fragment
**5**	Subchondral cyst formation

Hepple S, Winson IG, Glew D. Osteochondral lesions of the talus: a revised classification. Foot Ankle Int. 1999;20(12):789-93.

**Table 4:** CT staging system for osteochondral lesions of the talus.

**Stage**	**Definition**
**I**	Cystic lesion with intact roof
**IIA**	Cystic lesion with communication to talar dome surface
**IIB**	Open articular surface lesion with overlying nondisplaced fragment
**III**	Nondisplaced fragment with lucency
**IV**	Displaced fragment

CT classifcation of OLT - Ferkel RD, Sgaglione NA, DelPizzo W. Arthroscopic treatment of osteochondral lesions of the talus: long-term results. Orthop Trans. 1990; 14:172-8.

**Table 5:** Arthroscopic staging system for osteochondral lesions of the talus.

**Grade**	**Definition**
**A**	Smooth, intact, but soft cartilage
**B**	Rough cartilage
**C**	Fibrillations or fissures
**D**	Flap present or bone exposed
**E**	Loose, nondisplaced fragment
**F**	Displaced fragment

Cheng/Ferkel Arthroscopic staging system of OLT Cheng MS, Ferkel RD, Applegate GR. Osteochondral lesions of the talus: a radiologic and surgical comparison. Abstract presented at the Annual Meeting of the American Academy of Orthopedic Surgeons New Orleans. 1995.
